# Descemet stripping automated endothelial keratoplasty in Fuchs’ corneal endothelial dystrophy: anterior segment optical coherence tomography and in vivo confocal microscopy analysis

**DOI:** 10.1186/s12886-015-0096-x

**Published:** 2015-08-08

**Authors:** Rita Mencucci, Eleonora Favuzza, Ruggero Tartaro, Massimo Busin, Gianni Virgili

**Affiliations:** Eye Clinic, Department of Surgery and Translational Medicine, University of Florence, Florence, Italy; Department of Ophthalmology, ‘Villa Igea’ Hospital, Forlì, Italy

## Abstract

**Background:**

To evaluate the in vivo corneal changes using in vivo confocal microscopy (IVCM) and anterior segment optical coherence tomography (AS-OCT) in patients with Fuchs’ dystrophy who underwent Descemet stripping automated endothelial keratoplasty (DSAEK) and the relationship between these changes and the postoperative visual recovery up to 1-year follow-up.

**Methods:**

Before DSAEK and 1 day, 3, 6 and 12 months after surgery 31 patients (39 pseudophakic eyes) underwent a complete ophthalmological evaluation including best corrected visual acuity (BCVA), IVCM (subepithelial haze, interface haze, graft thickness) and AS-OCT (graft thickness).

**Results:**

Graft thickness measurements by AS-OCT were strongly correlated to those obtained using IVCM at every follow-up stage (intraclass correlation coefficient = 0.95 to 0.97 between 3 and 12 months, P < 0.001 for all coefficients). No correlation between BCVA and graft thickness measured by AS-OCT at any follow-up stage was found, while at 3 and 6 postoperative months the correlations between BCVA and preoperative subepithelial haze (r = 0.61, P < 0.001 and r = 0.46, *P* = 0.002), interface haze (r = 0.51, P < 0.001 and r = 0.46, *P* = 0.003), postoperative subepithelial haze (r = 0.43, *P* = 0.004 and r = 0.39, *P* = 0.001) were significant.

**Conclusions:**

The study confirmed corneal subepithelial haze and interface haze as important factors limiting visual acuity after DSAEK, while graft thickness was not related to BCVA.

## Background

Notwithstanding the growing interest on Descemet membrane endothelial keratoplasty (DMEK), Descemet stripping automated endothelial keratoplasty (DSAEK) is still one of the treatment of choice for Fuchs’ corneal endothelial dystrophy. This surgical technique allows the selective replacement of the posterior dysfunctional part of the cornea, including endothelium, Descemet membrane and a variable small amount of posterior stroma [1, 2].

This technique has shown several advantages over penetrating keratoplasty (PK): it has been reported to cause lower astigmatism, lower suture-related complications than PK [3–5], fewer high-order corneal aberrations [6] and a more rapid visual recovery [7, 8]. The majority of patients actually reach a best corrected visual acuity (BCVA) of 20/40 or better [3, 7, 8]. Nevertheless, even in presence of clear grafts, the percentage of patient achieving a BCVA of 20/20 is relatively small, and it is also well-known that DSAEK leads to a small hyperopic shift [9–11], residual corneal aberrations, glare and reduced contrast sensitivity compared to normal [4, 5, 12–17]. Therefore the debate whether surgical technique, donor lenticule preparation method, graft characteristics or recipient characteristics can affect DSAEK clinical outcome is still ongoing [13, 14, 18–20]. Specifically, previous studies analyzed possible correlations between postoperative visual acuity or visual quality and factors such as recipient corneal preoperative haze, recipient age, duration of the disease at the time of surgery, graft thickness, recipient corneal thickness and densitometry, often obtaining controversial results [9, 21–24].

Among the most used devices to evaluate these parameters are anterior segment Optical coherence tomography (AS-OCT) and in vivo confocal microscopy (IVCM).

AS-OCT is a diagnostic method widely used in ophthalmology and a non-invasive useful device in DSAEK follow-up. It allows to evaluate graft adhesion in the immediate postoperative period and to measure graft and corneal thickness [25, 26].

IVCM is a non-invasive diagnostic test that allows to acquire images of all the corneal layers (epithelium, Bowman’s membrane, stroma, Descemet membrane and endothelium) and of their cellular components (superficial and basal cells, nerve fibers, stromal keratocytes, endothelial cells) [27]. It is therefore used in the diagnosis and follow-up of various corneal diseases. Furthermore the IVCM is often performed during preoperative evaluations and postoperative follow-up of corneal transplants and refractive surgery [22–24, 28–30]. To our knowledge, there isn’t any report in literature evaluating DSAEK outcome with both AS-OCT and IVCM, analyzing possible correlations between preoperative or postoperative corneal and graft characteristics and visual outcome.

The purpose of our study was to evaluate the in vivo corneal changes using white-light IVCM and time domain AS-OCT in patients with Fuchs’ endothelial dystrophy who underwent DSAEK and the relationship between these changes and the postoperative visual recovery up to 1 year follow-up.

## Methods

This prospective cohort study involved 39 pseudophakic eyes of 31 patients who underwent DSAEK surgery due to Fuchs’ corneal endothelial dystrophy at the Eye clinic, Careggi University Hospital of Florence, Italy, between March 2011 and September 2012. The study followed the tenets of the Declaration of Helsinki and was approved by the local ethic committee (Careggi University Hospital of Florence). All patients signed a surgical informed consent form before DSAEK and an informed consent for the study.

Exclusion criteria for this study were concurrent eye pathology as glaucoma, macular/retinal diseases significantly affecting visual acuity, amblyopia, optic neuritis, uveitis, scleritis, ocular infection, pre-existing severe corneal scarring.

### Preoperative evaluation

At the preoperative visit all patients underwent a complete ophthalmological evaluation including best corrected visual acuity (BCVA, LogMAR), slit lamp biomicroscopy, intraocular pressure measurement, fundus ophthalmoscopy, white light IVCM (Confoscan 4, Nidek Technologies, Birmingham, UK) and time domain AS-OCT (Visante OCT, Carl Zeiss Meditec Inc, Dublin, California, USA).

IVCM was performed with a 40 X magnification and the z-ring adapter [31]. Lamp brightness was set at 80 in all examinations. Images obtained by Confoscan 4 were approximately 460x345 microns wide.

Before the exam, a drop of topical anesthetic (Benoxinate) was instilled. The patient was then asked to steadily fixate a target and IVCM was performed on the central cornea. The exam lasted approximately 3 min. At the end of the examination, an antibiotic eye drop (gentamicin 0.3 %) was administered.

The preoperative subepithelial haze was measured as the mean between the peak value of LRU (light reflectance units) in the subepithelial area and the values at 25 microns in the endothelial direction and 25 microns in the epithelial direction. Since at each examination Confoscan 4 performed three different scans of the cornea, three mean values, one for each scan, were obtained and averaged.

Two trained investigators performed the examination and one masked investigator selected and analyzed IVCM and AS-OCT scans.

### Surgical technique

All patients underwent DSAEK surgery alone.

All procedures were performed under monitored anesthesia with peribulbar block.

The posterior lamellar grafts were supplied from the Cornea Bank of Lucca (Italy), after having been cut by a microkeratome with a a 350-micron head (Moria SA, Antony, France).

They were trephinated by the surgeon with a Hessburg-Barron donor corneal punch (Barron Precision Instruments, LLC, Grand Blanc, Michigan USA) to a diameter of 8.25–8.75 mm. All grafts had an endothelial cell count of at least 2500 cells/mm^2^. Graft thickness provided by the Bank was recorded.

The anterior chamber of the eye was then entered through a 4 mm clear corneal incision, and in order to prevent the anterior chamber collapse a maintainer was used. Descemet membrane was stripped from the central 8-8.5 mm diameter. The rolled endothelial graft was inserted using a Busin glide (Moria Inc, Antony, France) and a small air bubble that was injected to lift the graft. After centering the graft, the anterior chamber was completely filled with an air bubble that after 10 min was reduced to about the 80 % of the size of the endothelial graft.

All surgical procedures were performed by the same skilled surgeon, RM.

### Postoperative follow-up

Patients were evaluated 1 day, 1 week, 1 month, 3, 6 and 12 months after surgery. Whereas each visit consisted of complete ophthalmic examinations, including BCVA and slit-lamp examination, at 3, 6 and 12 month visits AS-OCT (graft thickness measurement) and IVCM (graft thickness, interface haze and subepithelial haze evaluation) were also performed.

Graft thickness was measured by IVCM by assessing the distance from the endothelium to the change of reflectance that suggests the beginning of donor-recipient interface, then calculating the mean between the values obtained in each of the three scans performed by IVCM at each examination. The donor-recipient interface was identified as the area where a change in cell morphology and/or extracellular matrix was detected.

The donor-recipient interface haze, the characteristic gain in reflectance that may be observed at the border between the donor graft and the recipient cornea, was also evaluated by IVCM. It was measured as the mean between the peak value of LRU at the interface and the values at 25 microns in the endothelial direction and 25 microns in the epithelial direction; then we averaged the three mean values obtained, one for each scan.

Postoperative subepithelial haze was measured as already described in the preoperative evaluation section.

Graft thickness was measured by AS-OCT on a horizontal cross sectional image obtained at the anterior corneal vertex, using the software-imbedded flap-tool (high-resolution corneal scan mode).

### Statistical analysis

Linear mixed models were used to compare the change of the covariates of interest (logMAR BCVA, haze and thickness-related variables) at various follow-up times, accounting for correlated data using time as a random slope at the subject level. In order to simultaneously assess the correlation between covariates at different follow-up times, while accounting for individual correlation, structural equation modeling (SEM) of standardized variables was performed. Intraclass correlation coefficient (ICC) was used to compare graft thickness measured by means of AS-OCT and IVCM.

All analyses were carried out using Stata software (StataCorp 13.1, College Station, TX).

Results were expressed as mean and standard deviation (SD). A value of P < 0.05 was considered statistically significant.

## Results

The mean age of the patients was 73 ± 9.3 years (55 to 81 years).

**Fig. 1:**
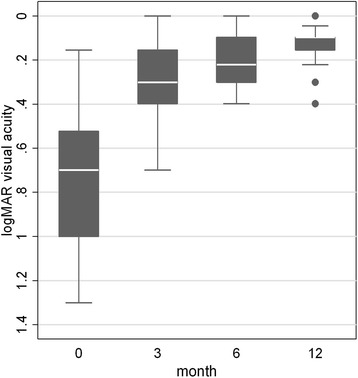
Best corrected visual acuity (BCVA) significant improvement up to 1-year after Descemet stripping automated endothelial keratoplasty for Fuchs’ corneal dystrophy (P < 0.001 at each postoperative visit vs preoperative value)

BCVA gradually improved during the follow-up (Fig. 1 and Table 1): the mean preoperative BCVA was 0.78 logMAR (20/120 Snellen) (SD = 0.35 logMAR) and significantly increased to 0.13 logMAR (20/25 Snellen) at 1 year (SD = 0.09 logMAR) (P < 0.001).

**Table 1:** Descemet stripping automated endothelial keratoplasty: main outcome measures

Outcome measure	Preoperative	3 months	6 months	1 year
BCVA (LogMAR)	0.78 (0.35)	0.28 (0.15)**	0.20 (0.11)**	0.14 (0.9)**
Graft Thickness (AS-OCT) (μm)	132.3 (32.7)	124.8 (34.0)*	117.8 (36.)**	111.0 (33.8)**
Graft Thickness (IVCM) (μm)	NA	130.0 (35.2)	120.7 (36.7)*	114.7 (37.4)*
Subepithelial haze (LRU)	133.0 (34.3)	98.1 (48.0)*	82.8 (33.9)*	75.6 (32.1)**
Interface haze (LRU)	NA	89.7 (49.3)	71.7 (37.1)*	68.8 (36.9)*

Results are expressed as mean (SD)

AS-OCT = Anterior segment optical coherence tomography

IVCM = In vivo confocal microscopy

*LRU* Light reflectance units

*NA* Not applicable

* = P <0.05 vs preoperative or 3-month (for graft thickness by IVCM and interface haze) value

** = P < 0.001 vs preoperative value or 3-month (for graft thickness by IVCM and interface haze) value

### Graft thickness

Mean graft thickness was 132.3 μ (SD = 32.7 μ) at baseline, as measured by the Eye Bank using Visante AS-OCT.

**Fig. 2:**
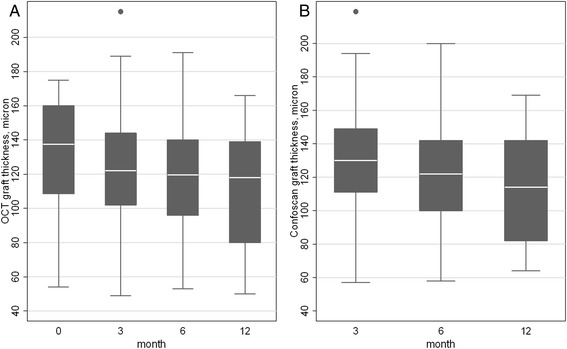
Graft thickness up to 1-year follow-up after Descemet stripping automated endothelial keratoplasty, measured by anterior segment-OCT (AS-OCT) and in vivo confocal microscopy (IVCM). **a** A statistically significant reduction of graft thickness measured by anterior segment-OCT compared to the preoperative value (*P* = 0.022 at 3 months, p < 0.001 at 6 and 12 months) and to the 3-month value (*P* = 0.069 at 6 months, p < 0.001 at 12 months) was observed. Preoperative graft thickness was provided by the Eye bank. **b** Postoperative graft thickness measured by in vivo confocal microscopy decreased significantly at 6 months and 1 year compared with the 3-month value (*P* = 0.026 and *P* = 0.003, respectively)

During follow-up (Fig. 2a and Table 1), a statistically significant reduction of graft thickness measured by AS-OCT compared to the Eye Bank value (*P* = 0.022 at 3 months, P < 0.001 at 6 and 12 months) was observed. In comparison with the 3-month value, graft thickness reduction was not significant at 6 months (*P* = 0.069), while it was significant at 1 year (P < 0.001).

Graft thickness measured by IVCM decreased significantly at 6 months and 1 year (Fig. 2b and Table 1) compared with the 3-month value (*P* = 0.026 and *P* = 0.003, respectively).

Graft thickness measurements by AS-OCT were strongly correlated to those obtained using IVCM at any follow-up stage (ICC = 0.95 to 0.97 between 3 and 12 months, P < 0.001 for all coefficients). IVCM yielded slightly but significantly thicker measures compared to AS-OCT at 3 months (5.2 μ, 95 % CI 2.6 to 7.8 μ), 6 months (4.1 μ, 95 % CI 2.7 to 5.5 μ) and 12 months (3.5 μ, 95%CI 1.3 to 5.7 μ).

**Fig. 3:**
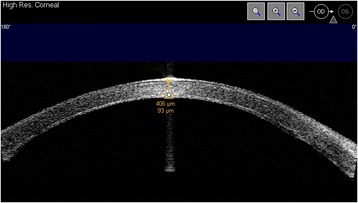
Graft thickness measured by AS-OCT 6 months after Descemet stripping automated endothelial keratoplasty. Central graft thickness (93 μ) was measured on a horizontal cross sectional image obtained at the corneal vertex, using the software-imbedded flap-tool (high-resolution corneal scan mode)

In Fig. 3 graft thickness measurement by AS-OCT is shown.

### IVCM corneal haze (subepithelial haze, interface haze)

Preoperative mean subepithelial haze was 133.0 LRU (SD = 33.3 LRU).

**Fig. 4:**
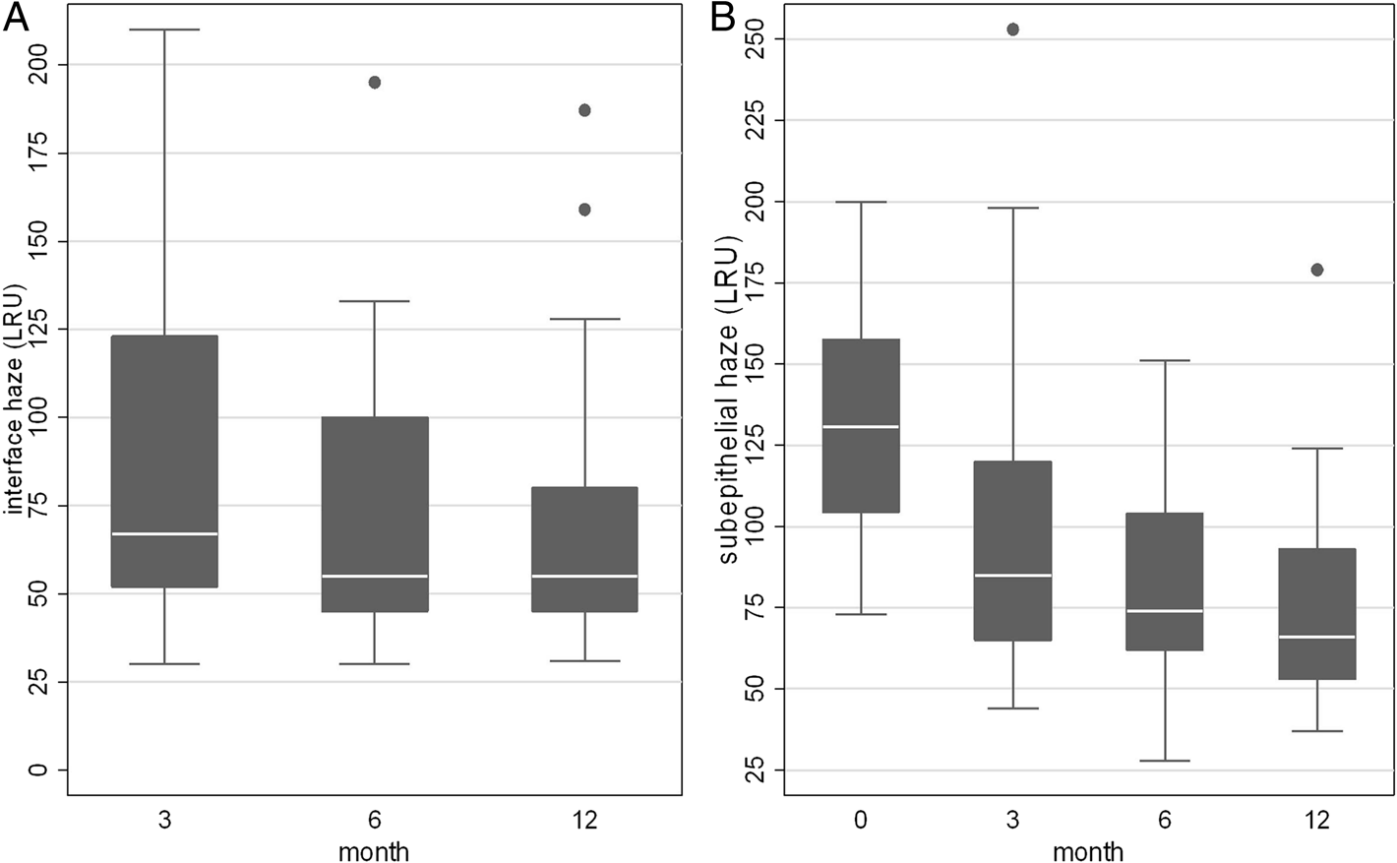
Postoperative donor-recipient interface haze and subepithelial haze measured by in vivo confocal microscopy. **a** At every follow-up stage donor recipient interface haze significantly decreased compared to the three month value (*P* = 0.006 at 6 months, *P* = 0.003 at 1 year). **b** Compared to the preoperative value (month 0) the reduction of subepithelial haze was significant at any follow-up stage (*P* = 0.013 at 3 months, *P* = 0.001 at 6 months, P < 0.001 at 1 year). LRU: light reflectance units

**Fig. 5:**
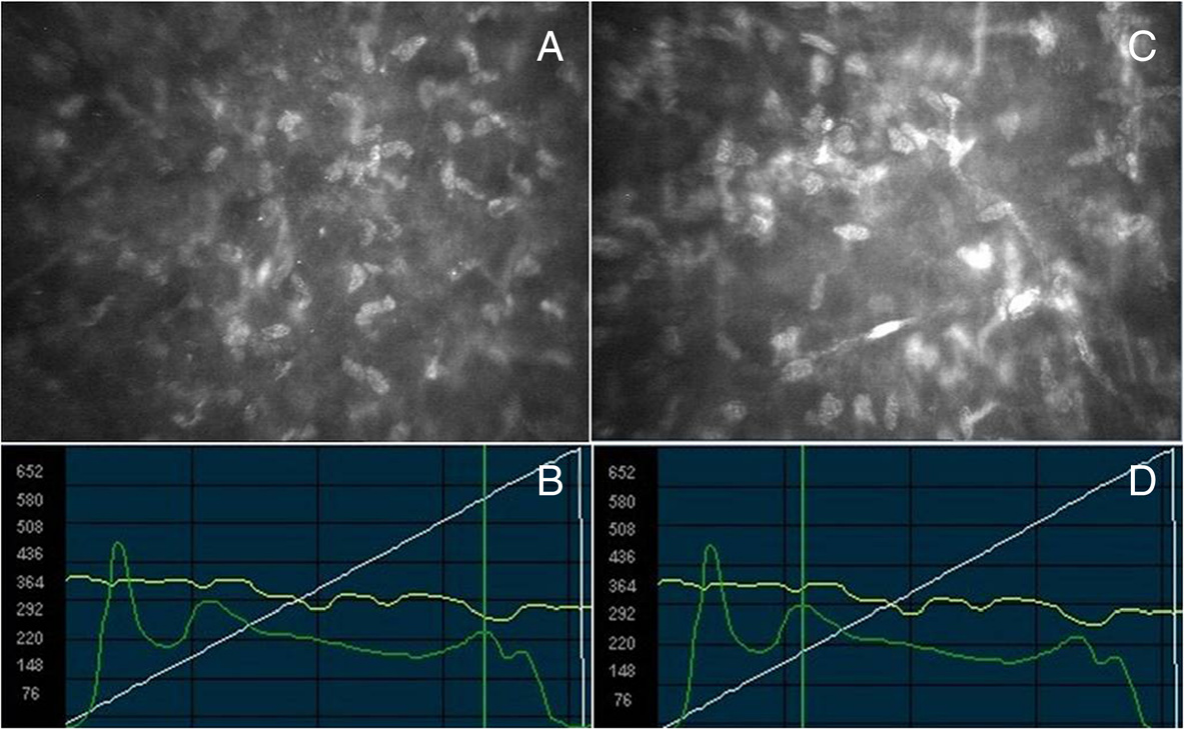
Confoscan images of the corneal subepithelial haze and the donor-recipient interface haze 6 months after Descemet stripping automated endothelial keratoplasty (DSAEK). **a** Corneal subepithelial scan, showing activated keratocytes and probably fibroblast. **b** Confoscan graph show the characteristic peaks of reflectivity that can be noted after DSAEK: from left to right the endothelial peak, the donor-recipient interface peak, the subepithelial and the epithelial peak. The cursor (vertical green line), corresponding to image (A), is located at the subepithelial reflectivity peak. **c** Graft interface scan of the same patient at the same visit. **d** Confoscan graph indicating the position of the scan (C), at the donor-recipient interface reflectivity peak

At every follow-up stage a statistically significant donor-recipient interface haze decrease compared to the three month value (Fig. 4a and Table 1) (*P* = 0.006 at 6 months, *P* = 0.003 at 1 year) were found, as well as a significant postoperative subepithelial haze reduction (*P* = 0.013 at 3 months, *P* = 0.001 at 6 months, P < 0.001 at 1 year compared to the preoperative value) (Fig. 4b and Table 1). In Fig. 5 IVCM scans of subepithelial haze and interface haze are shown.

### Correlations between best corrected visual acuity and other parameters

Even though graft thickness measured by AS-OCT and BCVA at any follow-up stage were not significantly correlated (r = -0.08, r = -0.01, r = -0.28 respectively; P > 0.05 for all coefficients), a modest correlation between the reduction of graft thickness and the improvement of BCVA between month 6 and month 12 (r = 0.39, *P* = 0.049) was observed.

**Fig. 6:**
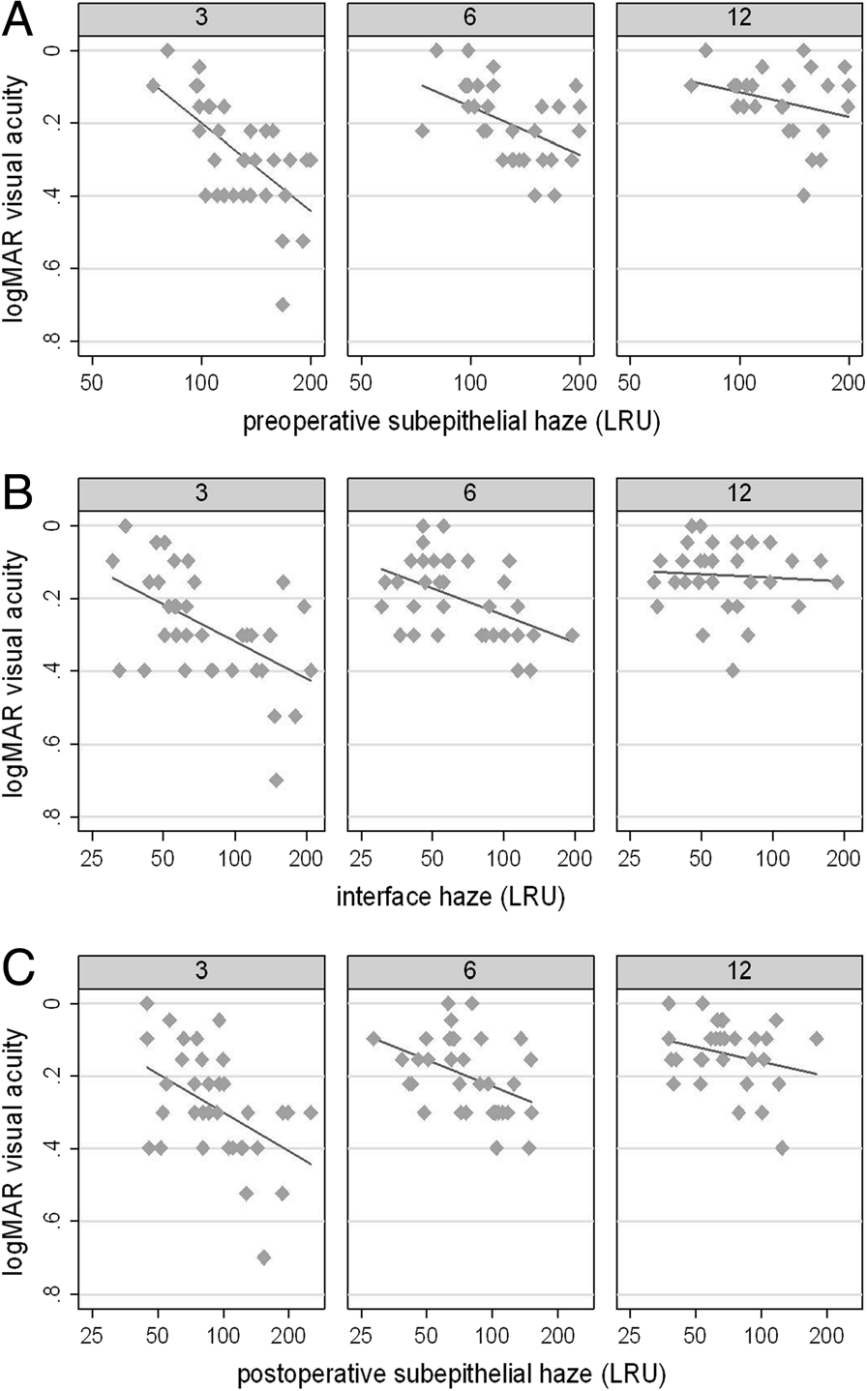
Relationship between postoperative best corrected visual acuity (BCVA) and preoperative subepithelial haze, interface haze, postoperative subepithelial haze at any follow-up visit after Descemet stripping automated endothelial keratoplasty. **a** A significant correlation between preoperative subepithelial haze and postoperative BCVA was found at 3 and 6 months (r = 0.61, P < 0.001 and r = 0.46, *P* = 0.002 respectively), while it was not significant at month 12 (r = 0.32, *P* = 0.068). **b** At 3 months and 6 months follow-up interface haze and postoperative BCVA were significantly correlated (r = 0.51, P < 0.001 at 3 months; r = 0.46, *P* = 0.003 at 6 months), whereas there was no correlation at 1 year (r = 0.07, *P* = 0.69). **c** At 3 months and 6 months a low even if statistically significant correlation between postoperative subepithelial haze and postoperative BCVA (r = 0.43, *P* = 0.004 at 3 months; r = 0.39, *P* = 0.001 at 6 months) was found, while at 1 year the correlation was not significant (r = 0.26, P > 0.05). LRU: light reflectance units

There was a significant correlation between preoperative subepithelial haze and BCVA at 3 and 6 months (r = 0.61, P < 0.001 and r = 0.46, *P* = 0.002 respectively), while it was no more significant at month 12 (r = 0.32, *P* = 0.068) (Fig. 6a).

At 3 months and 6 months follow-up interface haze and postoperative BCVA were significantly correlated (r = 0.51, P < 0.001 at 3 months; r = 0.46, *P* = 0.003 at 6 months), whereas there was no correlation at 1 year (r = 0.07, *P* = 0.69) (Fig. 6b).

At 3 months and 6 months we found a low even if statistically significant correlation between postoperative subepithelial haze and postoperative BCVA (r = 0.43, *P* = 0.004 at 3 months; r = 0.39, *P* = 0.001 at 6 months) (Fig. 6c), while at 1 year the correlation was not significant (r = 0.26, P > 0.05).

A correlation between the decrease of subepithelial haze and the improvement of BCVA between 6 months and 3 months (r = 0.49, P > 0.001) and between 12 and 6 months (r = 0.40, *P* = 0.008) was also observed.

Finally, we checked whether the interval change in BCVA at 3-6 months and 6–12 months was simultaneously related to that of graft thickness and subepithelial and interface haze. This confirmed that an improvement of BCVA was not associated with graft thickness change, whereas a significant association was found with a reduction of subepithelial haze both at 3–to–6 (*P* = 0.017) and at 6–to–12 months (*P* = 0.001). Interface haze reduction positively affected BCVA at 6-to-12 months (*P* = 0.013) only.

## Discussion

Our series of DSAEK performed in pseudophakic patients affected by Fuchs’ corneal endothelial dystrophy showed good results in terms of visual gain and postoperative corneal haze reduction. While we didn’t find any influence of the graft central thickness on postoperative BCVA, our analysis indicated a inverse correlation between subepithelial and donor-recipient interface haze and visual outcome.

Several factors have been considered to affect post-DSAEK visual outcome in clear grafts and in absence of ocular comorbidities. First of all, graft thickness has been hypothesized to negatively influence the visual gain after DSAEK [9, 32], this being supported by the promising initial results of new techniques such as DMEK [33, 34]. DMEK includes the implantation of thin grafts, composed only of Descemet membrane and endothelium, without donor stroma remnants. A discussion is still ongoing on this subject: many authors did not find any influence of a preoperative [20] or postoperative [17, 18, 21, 25, 35] thicker graft on postoperative BCVA. Even if we observed a statistically significant reduction of graft thickness during the 1 year follow-up, our results showed no correlation between visual outcome and postoperative central graft thickness, that was measured by both AS-OCT and IVCM.

Since to our best knowledge reports about a possible correspondence between the pachymetric measurements of the AS-OCT Visante and the IVCM Confoscan 4 are lacking, we report for the first time a good correlation between the graft thickness obtained by the two instruments, suggesting that IVCM can be considered a reliable instrument also for pachymetric measurements [31].

It has been reported [9, 14, 32] that other characteristics of the endothelial graft, such as the often not uniform thickness and the asymmetry of the graft, may more negatively affect the visual outcome after DSAEK. Graft prepared by a microkeratome often shows uneven thickness, with the center thinner than the periphery, and/or asymmetry: these factors are considered the main cause of the hyperopic shift observed after DSAEK and of posterior corneal high-order aberrations respectively [10, 32].

Another important factor that has been considered in affecting BCVA after DSAEK is corneal haze (or backscatter), that can be mainly located in the subepithelial region and in the graft-host stromal interface [22, 30, 36].

The subepithelial haze is a common finding to several corneal abnormalities, such as Fuchs’ endothelial dystrophy or other chronic endothelial dysfunction, and it has been related to tissue changes such as fibrosis caused by chronic edema [28, 36]; together with interface haze, it has been considered an expression of wound healing response that includes keratocyte activation and stromal extracellular matrix remodeling [30]. It has been demonstrated that it can persist for years after DSAEK, even though slowly decreasing, and the amount of both preoperative and postoperative haze has been related to low visual outcome after DSAEK [36]. The real clinical impact of subepithelial haze, whether it can affect visual acuity, visual quality or both it is still not clear: while some authors state that BCVA can be affected by subepithelial haze [22], others found no correlation with the visual acuity but only with visual quality in terms of forward light scatter or glare disability [23].

In our study we found a correlation between preoperative subepithelial haze and BCVA at 3 and 6 month follow-up, and between postoperative subepithelial haze and BCVA at the same visits. We found also a correlation between the change of postoperative subepithelial haze and that of BCVA between 6 months and 3 months and between 12 and 6 months, suggesting that it can be an important factor limiting DSAEK visual outcome.

Since the preoperative subepithelial haze can affect postoperative visual outcome (directly or causing more postoperative subepithelial haze), and a correlation between the amount of the subepithelial haze with the duration and the severity of corneal edema in the host cornea before surgery and between patients’ age and visual outcome has been found [23, 36], we agree with the hypothesis that could be crucial to perform DSAEK earlier in the natural history of the disease, possibly reducing the duration and the amount of the subepithelial fibrosis, before it become irreversible [23, 36].

Also donor-recipient interface haze has been assumed to affect visual outcome after endothelial keratoplasty [13, 37, 38]; in contrast Espana et al [30] and Baratz et al [23] did not find a significant correlation between interface reflectivity and visual outcome. Our data show a correlation between interface haze and postoperative BCVA at 3 and 6 month follow-up, whereas considering the interval changes interface haze reduction positively affected BCVA at 6-to-12 months only.

Several instruments has been recently used to measure corneal haze or backscatter, such as Scheimpflug Camera [10, 37] and AS-OCT [12]. Confoscan 4 IVCM, even if it may be more operator-dependent in comparison with other instruments and may require more patient compliance in order to obtain good quality scans and reproducible measurements, allows to assess in the same examination the characteristics of all the corneal layers at a cellular level and to correlate the scan peak representing corneal haze with cellular and tissutal alterations, giving more precise indication of their location. It can also perform cell count and, as previously stated, pachymetric measurements. It can provide quantitative information about corneal haze while giving an inner vision of the corneal tissue, useful in postoperative evaluation after corneal transplant. Drawbacks of this technique could be the small dimension of the area analyzed and the lack of a overall view of the cornea.

## Conclusions

Our study confirmed the subepithelial haze and the interface haze as important factors limiting visual acuity after DSAEK, while central graft thickness was not related to BCVA. No correlation between these parameters was found at 1 postoperative year, maybe due to the reduced range of visual acuity reached after the gradual improvement observed during the follow-up and to the limited number of patients.

Limitations of our study are the lack of the aberrometric evaluation of postoperative quality of vision and the short follow-up.

Further larger studies are necessary to investigate the role of corneal haze and other parameters in affecting not only visual acuity but also visual quality after DSAEK, and to assess their impact in patients’ quality of life after DSAEK.

## Abbreviations

IVCM: In vivo confocal microscopy
AS-OCT: Anterior segment optical coherence tomography
DSAEK: Descemet stripping automated endothelial keratoplasty
BCVA: Best corrected visual acuity
DMEK: Descemet membrane endothelial keratoplasty
PK: Penetrant keratoplasty
LRU: Light reflectance unit
SEM: Structural equation modeling
ICC: Intraclass correlation coefficient
SD: Standard deviation

## References

[1] Nanavaty MA, Wang X, Shortt AJ (2014). Endothelial keratoplasty versus penetrating keratoplasty for Fuchs endothelial dystrophy. Cochrane Database Syst Rev.

[2] Gorovoy MS (2006). Descemet-stripping automated endothelial keratoplasty. Cornea.

[3] Price FW, Price MO (2006). Descemet’s stripping with endothelial keratoplasty in 200 eyes: early challenges and techniques to enhance donor adherence. J Cataract Refract Surg.

[4] Koenig SB, Covert DJ, Dupps WJ, Meisler DM (2007). Visual acuity, refractive error, and endothelial cell density six months after Descemet stripping and automated endothelial keratoplasty (DSAEK). Cornea.

[5] Koenig SB, Covert DJ (2007). Early results of small-incision Descemet’s stripping and automated endothelial keratoplasty. Ophthalmology.

[6] Chamberlain W, Omid N, Lin A, Farid M, Gaster RN, Steinert RF (2012). Comparison of corneal surface higher-order aberrations after endothelial keratoplasty, femtosecond laser-assisted keratoplasty, and conventional penetrating keratoplasty. Cornea.

[7] Bahar I, Kaiserman I, McAllum P, Slomovic A, Rootman D (2008). Comparison of posterior lamellar keratoplasty techniques to penetrating keratoplasty. Ophthalmology.

[8] Chen ES, Terry MA, Shamie N, Hoar KL, Friend DJ (2008). Descemet-stripping automated endothelial keratoplasty: six-month results in a prospective study of 100 eyes. Cornea.

[9] Jun B, Kuo AN, Afshari NA, Carlson AN, Kim T (2009). Refractive change after Descemet stripping automated endothelial keratoplasty surgery and its correlation with graft thickness and diameter. Cornea.

[10] Scorcia V, Matteoni S, Scorcia GB, Scorcia G, Busin M (2009). Pentacam assessment of posterior lamellar grafts to explain hyperopization after Descemet’s stripping automated endothelial keratoplasty. Ophthalmology.

[11] Holz HA, Meyer JJ, Espandar L, Tabin GC, Mifflin MD, Moshirfar M (2008). Corneal profile analysis after Descemet stripping endothelial keratoplasty and its relationship to postoperative hyperopic shift. J Cataract Refract Surg.

[12] Hindman HB, Huxlin KR, Pantanelli SM, Callan CL, Sabesan R, Ching SS (2013). Post-DSAEK optical changes: a comprehensive prospective analysis on the role of ocular wavefront aberrations, haze, and corneal thickness. Cornea.

[13] Terry MA, Shamie N, Chen ES, Phillips PM, Hoar KL, Friend DJ (2009). Precut tissue for Descemet’s stripping automated endothelial keratoplasty: vision, astigmatism, and endothelial survival. Ophthalmology.

[14] Rao SK, Leung CK, Cheung CY, Afshari NA (2008). Descemet stripping endothelial keratoplasty: effect of the surgical procedure on corneal optics. Am J Ophthalmol.

[15] Hwang RY, Gauthier DJ, Wallace D, Afshari NA (2011). Refractive changes after Descemet stripping endothelial keratoplasty: a simplified mathematical model. Invest Ophthalmol Vis Sci.

[16] Muftuoglu O, Prasher P, Bowman RW, McCulley JP, Mootha VV (2010). Corneal higher-order aberrations after Descemet’s stripping automated endothelial keratoplasty. Ophthalmology.

[17] Seery LS, Nau CB, McLaren JW, Baratz KH, Patel SV (2011). Graft thickness, graft folds, and aberrations after Descemet stripping endothelial keratoplasty for fuchs dystrophy. Am J Ophthalmol.

[18] Phillips PM MD, Phillips LJ, Maloney CM (2013). Preoperative graft thickness measurements do not influence final BSCVA or speed of vision recovery after descemet stripping automated endothelial keratoplasty. Cornea.

[19] Price MO, Price FW (2006). Descemet’s stripping with endothelial keratoplasty: comparative outcomes with microkeratome dissected and manually dissected donor tissue. Ophthalmology.

[20] Daoud YJ, Munro AD, Delmonte DD, Stinnett S, Kim T, Carlson AN (2013). Effect of cornea donor graft thickness on the outcome of Descemet stripping automated endothelial keratoplasty surgery. Am J Ophthalmol.

[21] Ivarsen A, Hjortdal J (2014). Recipient corneal thickness and visual outcome after Descemet's stripping automated endothelial keratoplasty. Br J Ophthalmol.

[22] Kobayashi A, Mawatari Y, Yokogawa H, Sugiyama K (2008). In vivo laser confocal microscopy after Descemet stripping with automated endothelial keratoplasty. Am J Ophthalmol.

[23] Baratz KH, McLaren JW, Maguire LJ, Patel SV (2012). Corneal haze determined by confocal microscopy 2 years after Descemet stripping with endothelial keratoplasty for Fuchs corneal dystrophy. Arch Ophthalmol.

[24] Ahmed KA, McLaren JW, Baratz KH, Maguire LJ, Kittleson KM, Patel SV (2010). Host and graft thickness after Descemet stripping endothelial keratoplasty for Fuchs endothelial dystrophy. Am J Ophthalmol.

[25] Di Pascuale MA, Prasher P, Schlecte C, Arey M, Bowman RW, Cavanagh HD (2009). Corneal deturgescence after Descemet stripping automated endothelial keratoplasty evaluated by Visante anterior segment optical coherence tomography. Am J Ophthalmol.

[26] Yoo SH, Kymionis GD, Deobhakta AA, Ide T, Manns F, Culbertson WW (2008). One-year results and anterior segment optical coherence tomography findings of Descemet stripping automated endothelial Keratoplasty combined with phacoemulsification. Arch Ophthalmol.

[27] Pescosolido N, Komaiha C, Dapoto L, Lenarduzzi F, Nebbioso M (2012). Corneal haze in course of Fuchs’ endothelial dystrophy. Clin Ter.

[28] Mustonen RK, McDonald MB, Srivannaboon S, Tan AL, Doubrava MW, Kim CK (1998). In vivo confocal microscopy of Fuchs’ endothelial dystrophy. Cornea.

[29] Erie JC, McLaren JW, Patel SV (2009). Confocal microscopy in ophthalmology. Am J Ophthalmol.

[30] Espana EM, Huang B (2010). Confocal microscopy study of donor-recipient interface after Descemet’s stripping with endothelial keratoplasty. Br J Ophthalmol.

[31] McLaren JW, Nau CB, Patel SV, Bourne WM (2007). Measuring corneal thickness with the ConfoScan 4 and z-ring adapter. Eye Contact Lens.

[32] Dickman MM, Cheng YY, Berendschot TT, van den Biggelaar FJ, Nuijts RM (2013). Effects of graft thickness and asymmetry on visual gain and aberrations after Descemet stripping automated endothelial keratoplasty. JAMA Ophthalmol.

[33] Dapena I, Ham L, Melles GRJ (2009). Endothelial keratoplasty: DSEK/DSAEK or DMEK - the thinner the better?. Curr Opin Ophthalmol.

[34] Van Dijk K, Ham L, Tse WHW, Liarakos VS, Quilendrino R, Yeh RY (2013). Near complete visual recovery and refractive stability in modern corneal transplantation: Descemet membrane endothelial keratoplasty (DMEK). Contact Lens Anterior Eye.

[35] Shinton AJ, Tsatsos M, Konstantopoulos A, Goverdhan S, Elsahn AF, Anderson DF (2012). Impact of graft thickness on visual acuity after Descemet’s stripping endothelial keratoplasty. Br J Ophthalmol.

[36] Patel SV, McLaren JW (2013). In vivo confocal microscopy of Fuchs endothelial dystrophy before and after endothelial keratoplasty. JAMA Ophthalmol.

[37] Heinzelmann S, Böhringer D, Maier PC, Reinhard T (2014). Correlation between visual acuity and interface reflectivity measured by pentacam following DSAEK. Acta Ophthalmol.

[38] Ferrari G, Reichegger V, Ludergnani L, Delfini E, Macaluso C (2012). In vivo evaluation of DSAEK interface with scanning-laser confocal microscopy. BMC Ophthalmol.

